# The Diagnosis and Management of Pediatric Spine Infections

**DOI:** 10.7759/cureus.16748

**Published:** 2021-07-30

**Authors:** Ehab S Saleh, Christopher C Vasileff, Abdullah M Omari, Jad G Khalil

**Affiliations:** 1 Department of Orthopedics, Oakland University William Beaumont School of Medicine, Rochester, USA; 2 Department of Orthopedics, William Beaumont Hospital, Taylor, Taylor, USA

**Keywords:** ct-guided biopsy, spondylodiscitis, vertebral osteomyelitis, tubercular osteomyelitis, epidural abscess

## Abstract

The management of pediatric spine infections requires a multidisciplinary approach that includes orthopedic surgeons, infectious disease specialists, interventional radiologists, and others.

The prevalence of the disease has increased in frequency, virulence, and degree of soft tissue involvement over the past several years; there has also been a resurgence of some types of infections, such as tuberculosis, fungal, and viral pathogens.

The diagnosis can often be reached with a detailed history, physical examination, laboratory tests, and imaging studies. Pathologies mimicking infection require a more invasive approach for diagnosis, including core or open biopsy.

The treatment of discitis, spondylodiscitis, vertebral osteomyelitis, spinal epidural, and intramedullary abscesses in children is at times complex, and although many infections can be treated non-surgically with antibiotic therapy, some more extensive infections require surgical management.

A timely diagnosis is important as it allows the initiation of the appropriate antimicrobial therapy and would decrease the complexity of the subsequent surgical intervention.

## Introduction and background

Spinal infection in children includes infection of the spinal cord, the nerve roots, meninges, the vertebra, the intervertebral disc, the epidural, intradural, and intrathecal space, and post-operative infections [1]. The incidence of spinal infection in patients less than 20 years old living in developed countries is 0.3 per 100,000 [1].

The most common organism causing pyogenic infection of the spine in children is methicillin-sensitive *Staphylococcus aureus,* with *Kingella kingae *being especially common in patients between six months and four years of age [2,3].

Erythrocyte sedimentation rate was found to be more sensitive than C-reactive protein in aiding with the diagnosis in one cohort (78% versus 38%) [4].

Pediatric spinal infections have a triphasic age distribution; the first is in early infancy, the second is between six months and four years, and the third is in school-aged children [5]. The mean age at presentation is 4.3 years (range is from one to 15 years), and the female to male ratio is 1.3:1 [4].

In the pre-antibiotic era, pediatric spinal infections had a 90% mortality rate, which has dropped to less than 5%, still a significant number. If the spinal infection is missed or treated late, this can lead to spinal deformities, instabilities, and neurologic complications [5].

Several factors pertaining to the pediatric population can make the diagnosis and treatment more challenging; these include difficulty obtaining a history in non-verbal children, low sensitivity of blood cultures, CT-guided biopsy, non-specific findings of imaging studies in the early stages of the disease, and the lack of consensus on the best treatment approach [4,5].

## Review

Anatomical considerations

The presence of pure discitis in children has been extensively debated among researchers, but the current available evidence indicates that the nucleus pulposus is avascular in both children and adults, and the cartilage endplate and annulus fibrosus receive variable blood supply though life. Pyogenic spondylitis in both age groups is a process that starts in the vertebral endplate [4]. Some similarities and differences of spondylodiscitis in adults and children are summarized in Table 1 [2,6].

**Table 1:** A comparison between pediatric and adult spondylodiscitis

	Pediatric	Adult
The initial seeding focus of the infection in the spine	Vertebral endplate	Vertebral endplate
The incidence of the infection	0.3 per 100,000 cases per year in persons less than 20 years	The overall incidence of spinal infections across all ages per year is 2.4 per 100,000, This incidence Increases with age to 6.5 per 100,000 per year in the 50-70 years of age group
Vascularity of the disc	Avascular nucleus pulposus, with variable vascularity of the endplate and the annulus fibrosus in children and adults through life	The same
Delay in diagnosis	Common	Common
Most common organism	Staphylococcus aureus	Staphylococcus aureus
Common source of infection	Subtle bacteremia from an ear, nose, and throat infection	Obvious bacteremia from intravenous drug use, urinary tract infection, and other comorbidities
Treatment	Mostly intravenous antibiotics with surgery occasionally required	Mostly intravenous antibiotics with surgery occasionally required
Comorbidities	Not common	Common
CT-guided biopsy	Not routinely done, only needed in chronic cases, atypical cases, and cases not clinically responding to antibiotics	Not routinely done, only needed in chronic cases, atypical cases, and cases not clinically responding to antibiotics

A study that retrospectively evaluated 103 children with spinal infection over a 10-year period found that none presented with only discitis. Spondylodiscitis (infection of the disc and vertebral body) was the primary presentation for toddlers, and vertebral osteomyelitis affected older children and adolescent more frequently. They concluded that pure discitis does not occur, and that a pyogenic infection will start at the metaphyseal region of the vertebra and then spread to adjacent vertebra and the intervening disc space [2].

A unique anatomical feature of the spine, which might lead to multilevel vertebral infection, is the presence of the dural space that is filled with fat and the valveless Batson’s venous plexus. Both the venous plexus and the fat may form a route for the dissemination of infection between multiple levels [6].

Pathogenesis

The usual route of spinal infection in the pediatric population is through a transient minor hematogenous bacteremia [4]. It can also be due to direct inoculation from a diagnostic or surgical procedure, following trauma or direct inoculation from an adjacent genitourinary, gastrointestinal, or oropharyngeal infection [5,7].

Pediatric blunt trauma or hyperextension cervical spine injury has been reported to be a causative factor in delayed cervical spine osteomyelitis, due to a possible small pharyngeal perforation that seeds the bone and eventually develops into osteomyelitis [8].

Spinal infections that happen as a complication of intrathecal baclofen treatment in children with cerebral palsy highly correlate with a gross motor function classification system level five, the presence of gastrostomy tubes, and a history of seizures [9].

Five cases of cervical spine spondylodiscitis have been reported following button battery ingestion in children less than five years old, and the battery gets lodged in the esophagus, which leads to local injury, esophageal pressure necrosis, and perforation. The presentation is usually within one to two weeks of the foreign body removal. All reported cases were treated with intravenous antibiotics [10].

Pediatric spine infections can be pyogenic, like vertebral osteomyelitis and spondylodiscitis, or non-pyogenic, like parasitic, fungal, and tuberculous infection of the spine [1]. Methicillin-sensitive and methicillin-resistant Staph are the most common pathogens [1,2]. Other bacterial pathogens include *Kingella kingae*, group B streptococci, *Escherichia coli*, *Listeria *species, *Haemophilus influenzae*, *Neisseria meningitides*, and *Brucella *species [1,2].

*Bartonella henselae*, the causative bacteria of cat scratch disease, is a rare cause of multifocal osteomyelitis involving the spine and pelvis [11].

Lemierre disease, a rare cause of pediatric spondylodiscitis and epidural abscess, is caused by the anaerobe *Fusobacterium necrophorum*. These bacteria infect the pharynx initially and then spread to the spine through metastatic abscess formation [12].

Viral pathogens include the herpes virus, poliovirus, and cytomegalovirus [1]. Anogenital Herpes simplex virus (HSV) infection in adolescent patients can cause sacral myeloradiculitis that presents with acute urinary retention, constipation, perineal paresthesia, and erectile dysfunction in the setting of a genital herpes infection [7].

Fungal pathogens include *Aspergillus *and *Candida *species [1,13]. Parasitic causes include cysticercosis, schistosomiasis, toxoplasmosis, and echinococcal disease [1,14].

In sickle cell anemia patients, the most common causative organisms are *Staphylococcus aureus* and salmonella; but vaso-occlusive crisis should always be on the differential diagnosis of a sickle cell child presenting with musculoskeletal pain as there is a low incidence of osteoarticular bacterial infection in this category of children (1.6%) [15].

Diagnosis

a. History and Physical Examination

A triphasic distribution of primary spinal infections exists with 79% of cases presenting between the ages of six months and four years, 20% in the juvenile and adolescent age group, and 1% in children under six months of age [2].

Pediatric spondylodiscitis symptoms depend on the age of the child, with infants presenting with irritability and toddlers presenting with a limp, refusal to sit or walk, and abdominal pain; 75% will not have fever. Older children might present with back pain and point tenderness [4].

The diagnosis is often delayed (two days to 11.7 months with a mean of 27 days) [2].

b. Laboratory Analysis

The white blood cell (WBC) count and C-reactive protein (CRP) are mostly normal. The erythrocyte sedimentation rate (ESR) is usually increased to more than 50 mm/hour in 94% of the cases [4]. Analysis of serial ESR measurements confirmed this test to be the most accurate for plotting the clinical course of the condition [1,16,17].

Blood cultures in children with pyogenic spine infection are usually negative in the indolent illness presenting with vague back pain and positive in the acute febrile cases, and when they are positive, *S. aureus *is the most common organism isolated [16,18].

c. Imaging

**Figure 1:**
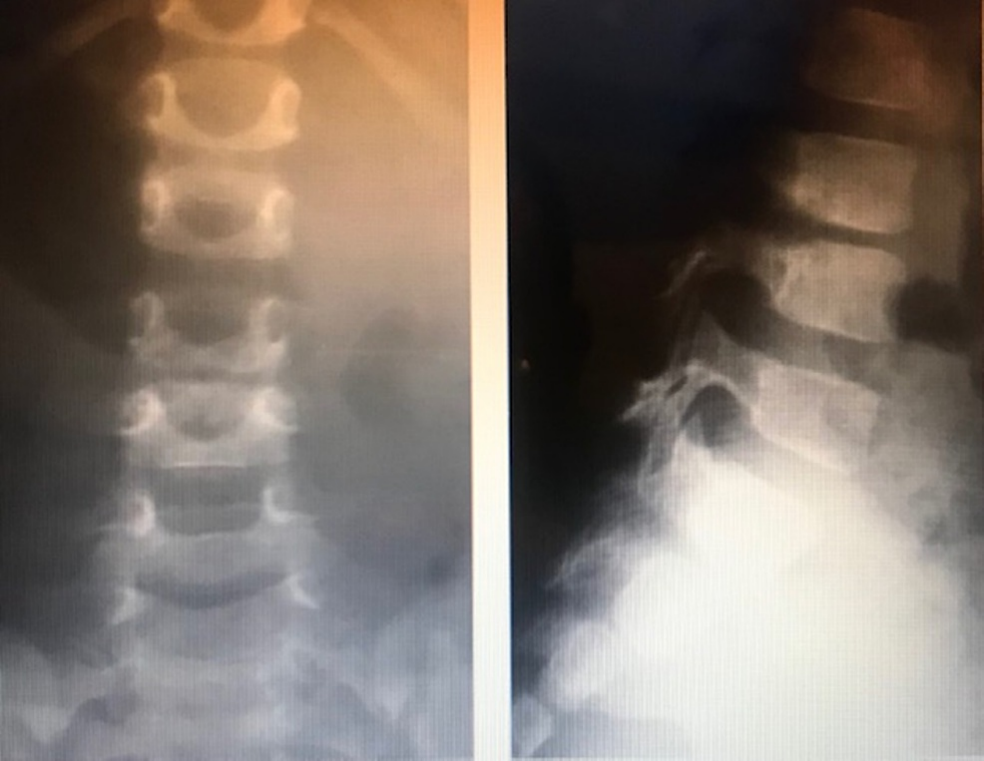
Lumbar spine x-ray of a three-year-old child with a two-week history of irritability, showing disk space narrowing of L3-L4 consistent with spondylodiscitis

**Figure 2:**
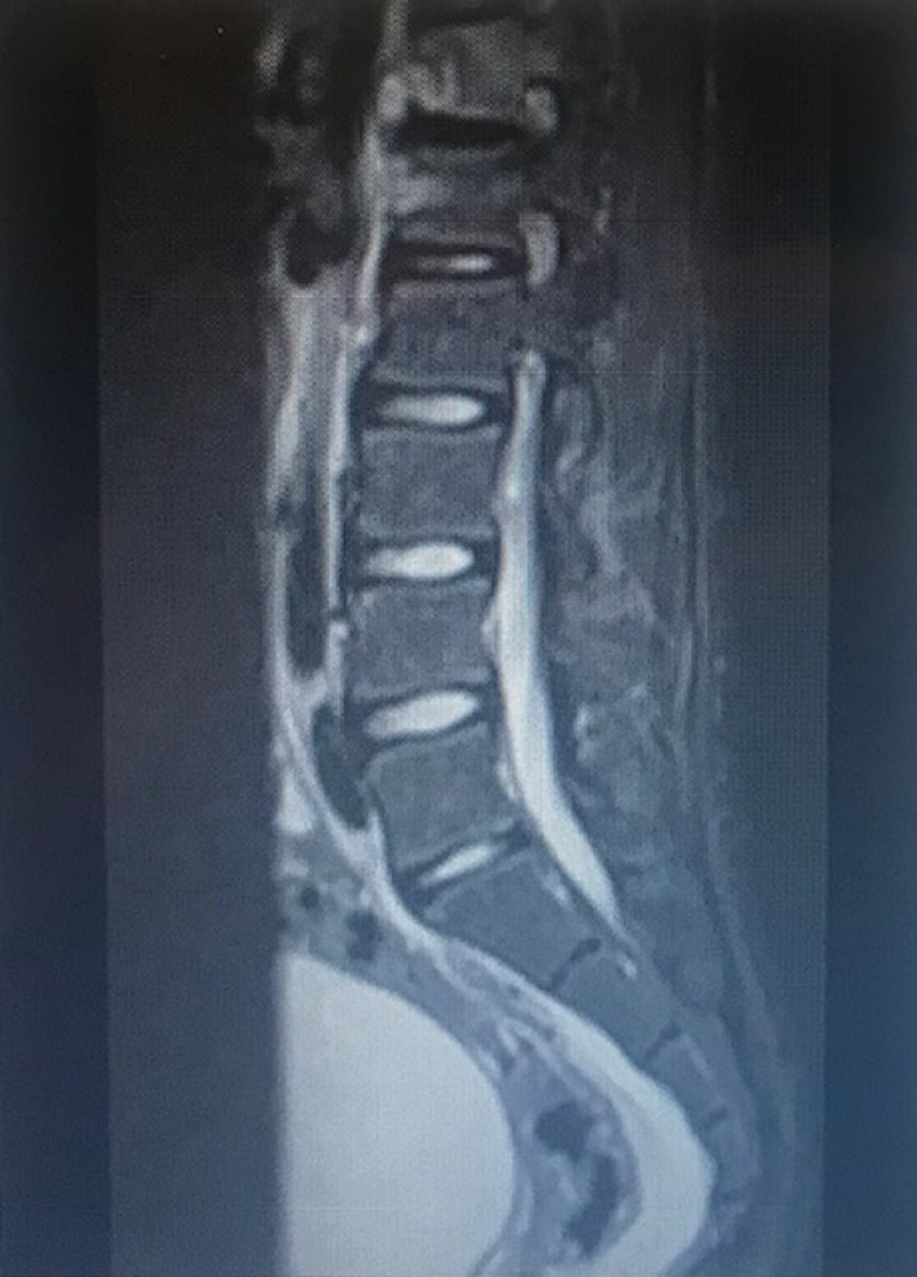
MRI of the lumbar spine of a 14-year-old child with spondylodiscitis involving the T12 and L1 vertebras and disc MRI, Magnetic resonance imaging.

**Figure 3:**
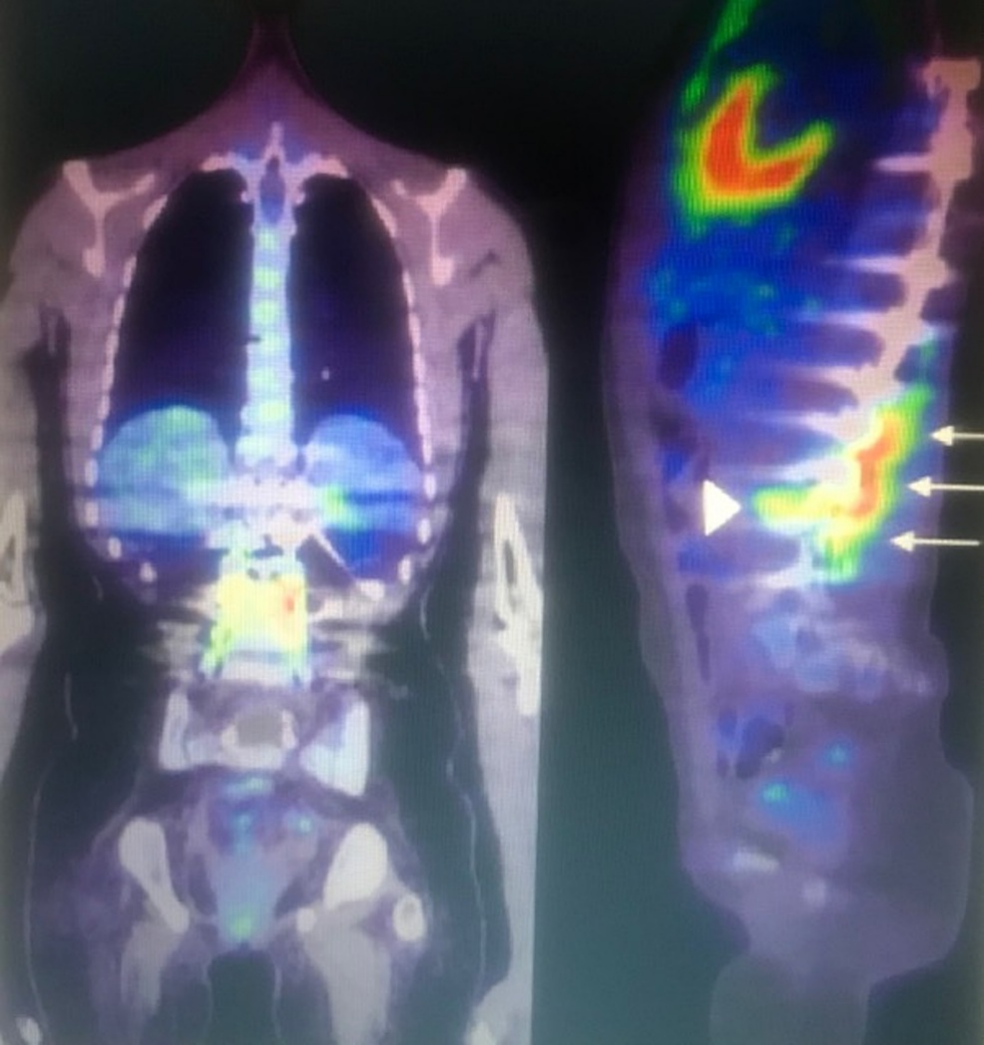
Thoracolumbar spinal hardware infection in a 14-year-old child. Coronal and sagittal 18f-FDG PET/CT views of the spinal hardware extending from T6 to L4. There is increased FDG uptake in the bone and soft tissues immediately adjacent to the hardware from T12 to L4 (arrows). FDG uptake at the bone hardware interface is present at the left L3 interpedicular screw (arrowheads). FDG, Fluorodeoxyglucose; PET, positron emission tomography; CT, computed tomography.

1. X-ray: This is not a sensitive study to diagnose spondylodiscitis; it can remain normal for two to eight weeks (Figure 1). X-ray findings can include irregularity of the vertebral endplate, disc space narrowing, and lack of definition of the vertebral endplates. In untreated cases, bony sclerosis may begin to appear in 10-12 weeks [19].

2. MRI: For the diagnosis of pediatric spine infections, MRI is more sensitive than x-rays or CT scan and more specific than bone scans. Its sensitivity is 96%, and specificity is 93% for an accuracy of 94%. An infection will show as a region of low T1 and high T2 signal intensity, with post-contrast enhancement on fat-suppressed T1-weighted images (Figure 2) [20,21].

3. 18F-FDG PET/CT: Fluorodeoxyglucose (FDG)-positron emission tomography (PET) CT scan is helpful in diagnosing spine infection when there is retained hardware, where an MRI will be affected by the metal artifact; it can also diagnose other hardware complications not related to infection and other extraspinal sources of infection (Figure 3) [22]. It is superior to Gallium-67 citrate and Technetium-99m bone scan and may be superior to MRI for detection of early or low-grade spondylodiscitis [21].

4. CT-guided biopsy: Spinal biopsy for cultures is not routinely done, and therapy is usually guided by clinical and radiological findings, but in chronic cases, atypical cases, and cases not clinically responding to IV antibiotic, a CT-guided biopsy can yield an organism if blood cultures were negative, which can determine the activity of the disease and guide treatment [18]. The success rate of CT-guided biopsy in finding an organism is between 36% and 91% [1], compared to a yield of 76% for open surgical biopsy [21].

An adult study to evaluate the impact of CT-guided biopsy on patients with suspected spondylodiscitis concluded that an initial CT-guided biopsy will yield a positive culture in about one-third of the cases; 75% of those initial positive cultures were useful in tailoring the treatment, but 96% of the study patients without atypical course and with negative blood cultures would have been successfully treated with an empirical course of antibiotic, without needing the CT-guided biopsy result [23].

Clinical Categories

a. Childhood Spondylodiscitis

Spondylodiscitis is the most common term used to describe primary pyogenic spinal infections that include discitis, spondylodiscitis, and vertebral osteomyelitis. Childhood spondylodiscitis has three clinical forms: the neonatal form (under six months), the infantile form (six months to four years), and a third form affecting children over four years of age including adolescents [2].

Spondylodiscitis seems to be the primary pathology in toddlers, whereas vertebral osteomyelitis affects older children and adolescents more [2,24].

b. Tuberculous Infection of the Spine

**Figure 4:**
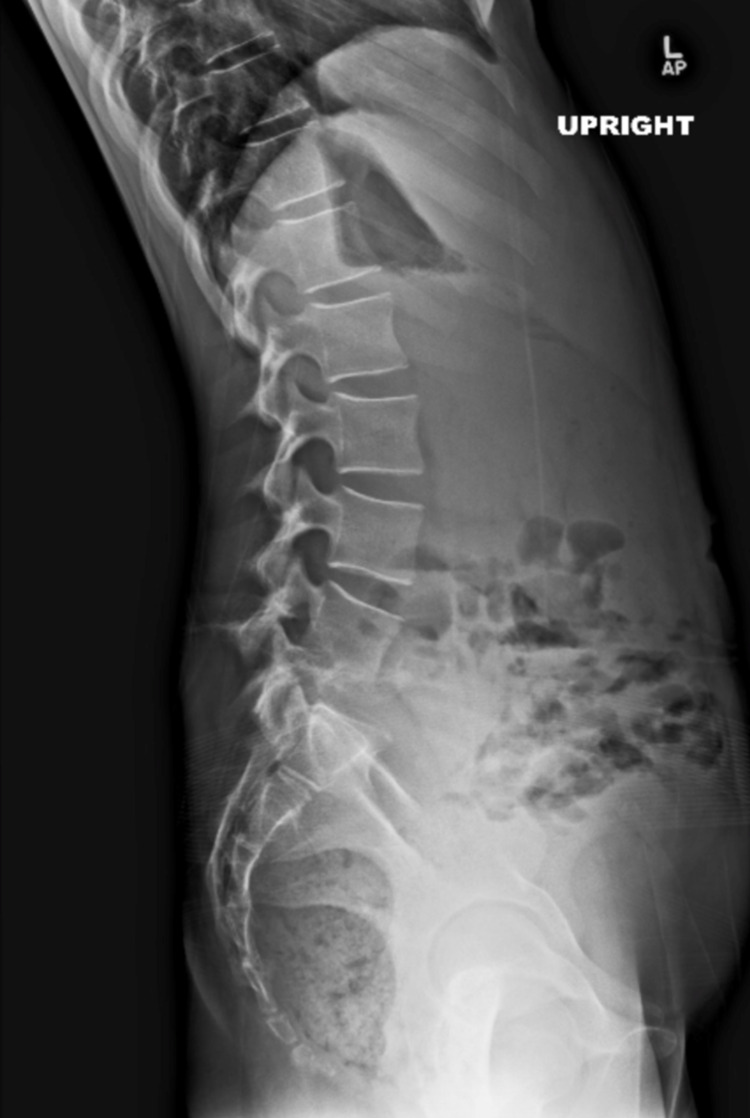
A lateral lumbar spine x-ray of a 12-year-old female The image shows a lateral lumbar spine x-ray of a 12-year-old female that presented to our institution with a four-month history of back pain, showing loss of the disc space and junctional kyphosis at L4/L5. She was diagnosed with tuberculosis of the lumbar spine.

Tuberculosis of the spine continues to be a problem in many parts of the world, and the spine is involved in 50% of cases of musculoskeletal tuberculosis, usually presenting with kyphosis, and neurological deficit (Figure 4) [25].

Children tend to have a more severe and complicated course than adults, and it remains to be a leading cause of pediatric paraplegia in developing countries [26]. But generally, paraplegia is rare in children, compared to adult patients with spinal tuberculosis [1].

Conventional tuberculosis cultures will produce results in four to six weeks, and they have a low yield. A better and faster way of laboratory diagnosis of tuberculosis is with nucleic acid amplification test, which will detect mycobacterial genes after gene amplification via a polymerase chain reaction [26].

**Figure 5:**
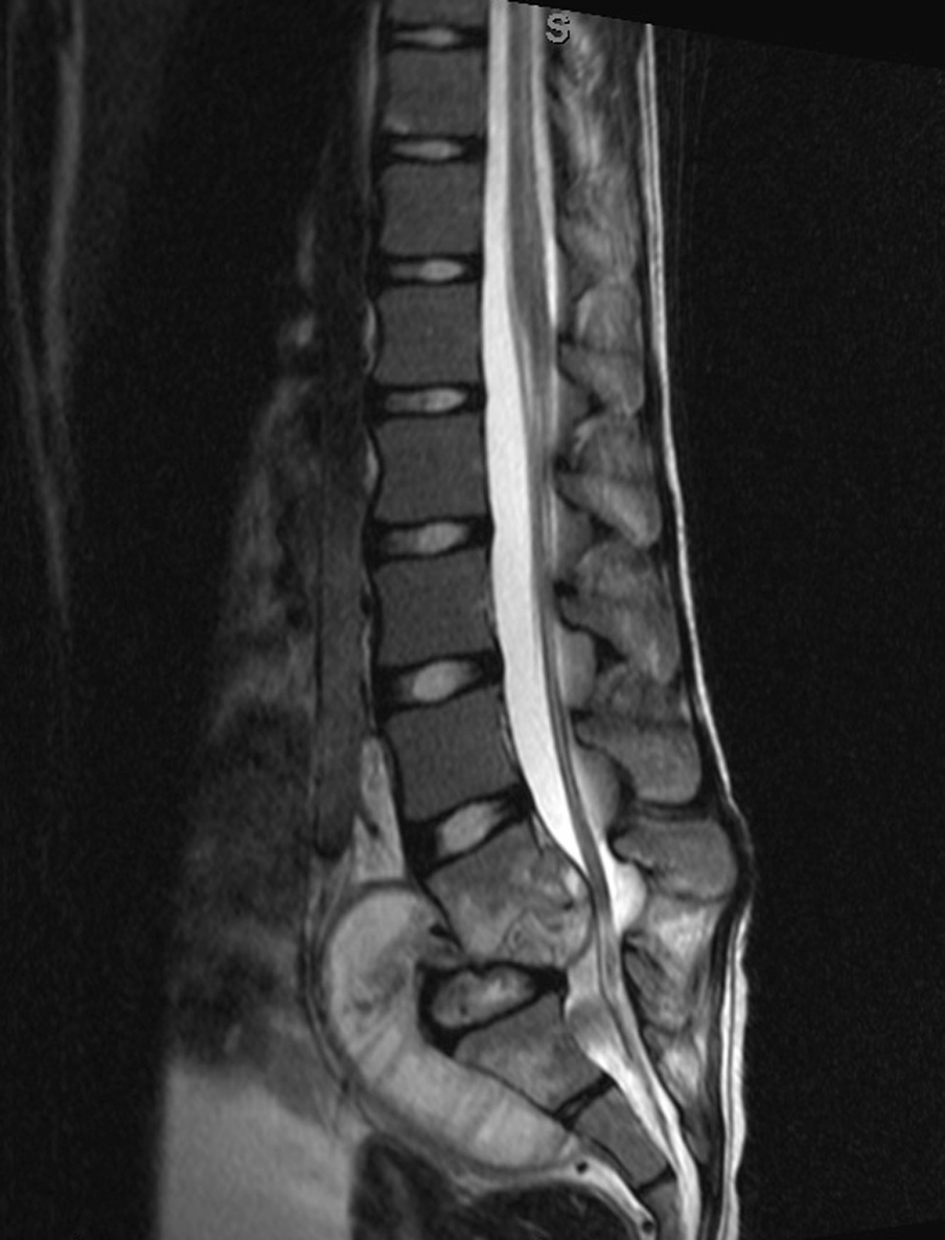
Sagittal MRI image of the lumbar spine of the same 12-year-old female with tuberculosis, showing multilevel involvement from L4 to S1 and a large anterior phlegmon

On MRI, the presence of multiple vertebral levels involvement with intervening normal levels (skip lesions) is suggestive of tuberculosis over pyogenic spondylodiscitis (Figure 5) [21].

Spinal tuberculosis without structural damage is generally treated with antibiotics and does not usually require surgery if diagnosed and treated early [27].

Indications for surgery include neurological deficits, spinal instability, severe and progressive kyphosis, lack of response to antitubercular agents, and a large paraspinal abscess [25].

**Figure 6:**
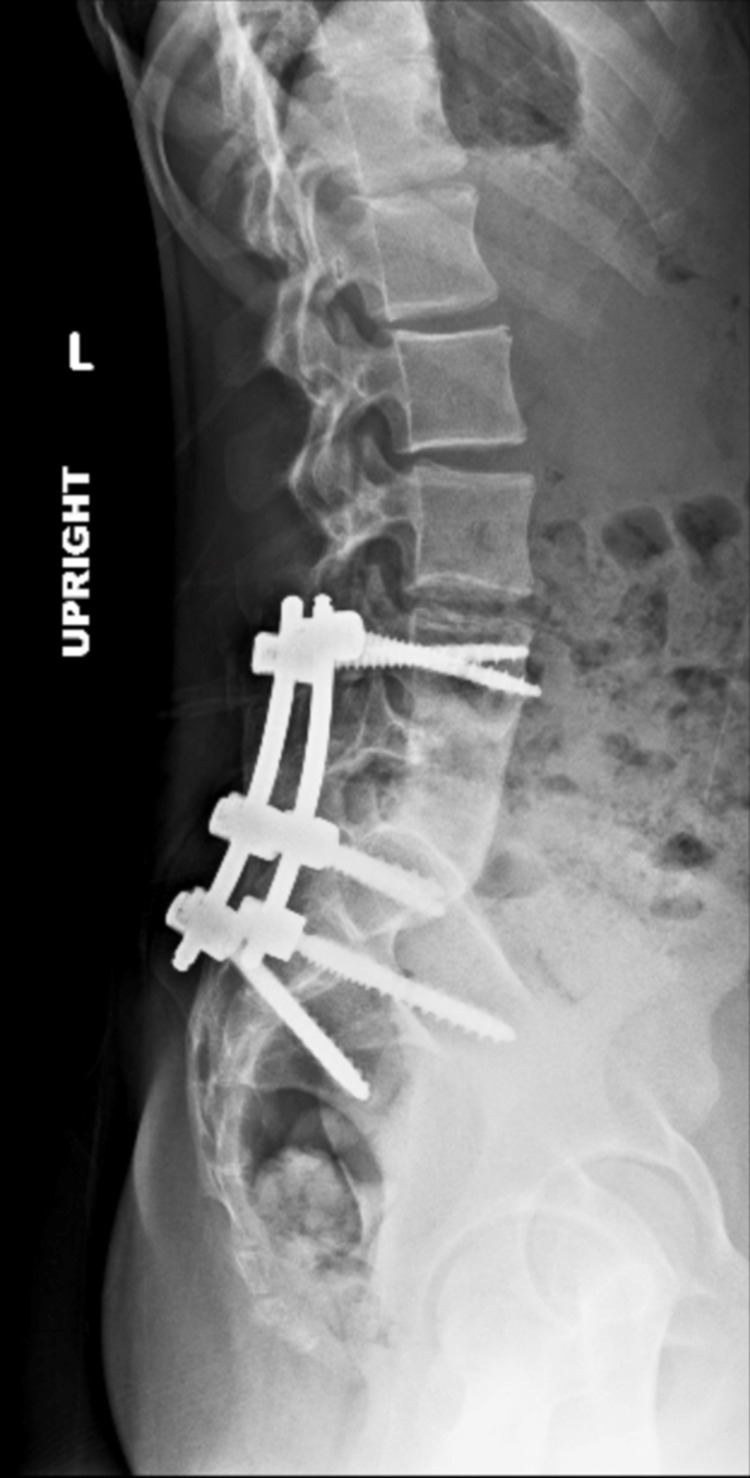
Image of the same 12-year-old girl with tuberculosis, after anterior L5 vertebrectomy, lumbar abscess debridement, anterior L4-S1 arthrodesis using tricortical allograft, and posterolateral arthrodesis of L4-S1

Multiple authors reported their good results with surgical treatment using anterior decompression and bone grafting, with posterior instrumentation and fusion for cases with neurological deficits not responding to three weeks of antibiotics therapy, and cases with progressive spinal instability and kyphosis (Figure 6) [25-27].

c. Cat Scratch Disease

Cat scratch disease is caused by *Bartonella henselae*, a gram-negative bacillus [28]; 50% of osteomyelitis caused by cat scratch disease involves the spine. Serology is the main microbiological diagnostic test, and it is mostly treated with antibiotics alone, and 20% of patients might require surgery with an overall good prognosis [29].

A series of five cases of pediatric cervical spine osteomyelitis caused by *Bartonella henselae* have been reported in the literature in which four of them were treated with antibiotics and surgery, and one was treated with antibiotics alone. The cases that were managed surgically required discectomy and fusion, laminectomy and drainage, anterior open biopsy and drainage, and traction followed by surgical drainage [28].

 *d. Spinal Epidural Abscess*

Childhood spondylodiscitis can be complicated with soft tissue abscess [2]. Earlier reports on pediatric spinal epidural abscess recommended early surgical drainage as the treatment of choice for all patients [30]. More recent reports did show that pediatric spinal epidural abscesses can be treated successfully with antibiotics alone or in combination with CT-guided drainage, with only a few needing formal surgical drainage [31,32].

Only 8% of pediatric patients with spinal epidural abscess present with the classic triad of fever, back pain, and neurologic deficit, and 33%-65% have no risk factors [32]. *Staphylococcus aureus* is the most frequently isolated organism [30-32]. As mentioned earlier, Lemierre disease is a rare cause of pediatric spondylodiscitis and epidural abscess formation [12].

e. Spinal Subdural Infection

Spinal subdural infection is the least common infection in the central nervous system. A total of 53% of spinal subdural infections in children were associated with spinal dysraphism and other congenital abnormalities of the spine. The most common organism internationally to infect the spinal subdural space in children is *Mycobacterium tuberculosis* followed by *Echinococcus granulosus*, *Staphylococcus* species, and *Streptococcus *species. The disease is usually treated surgically, although a more expectant approach consisting of antibiotics and observation has also been proposed [33].

f. Intramedullary Spinal Cord Abscess

This is a rare entity with less than 50 pediatric cases reported since 1830. In children, the most common cause is a congenital dermal sinus with acute cases presenting with fever, pain, and neurological deficit, whereas chronic cases present as an intramedullary tumor. Surgical treatment with multilevel laminectomy, myelotomy, and drainage of the abscess and six to 12 weeks of antibiotics is the treatment of choice, with 60% good-to-excellent result [34,35].

g. Spine Candida Infection

In children, the vertebra is the third most common site affected with candida osteomyelitis after the femur and humerus; inflammatory markers are mildly elevated or normal, and most patients will have two or more infected bones [13]. Because of the chronic nature and the multiple bone involvement characteristics of candida musculoskeletal infections, it can be confused with metastatic cancer and chronic multifocal bacterial osteomyelitis [13]. Advanced imaging studies can show skip lesions on MRI-like tuberculosis [21].

About half can be treated with antifungal agents only and the other half with surgical treatment and antifungal agents, given for six to 12 months, and a minority can be treated with only surgery [13].

h. Parasitic Infections

Four parasitic infections can involve the spine, cysticercosis, schistosomiasis, toxoplasmosis, and echinococcal disease. Spinal neurocysticercosis is rare, present in 1% to 6% of patients diagnosed with neurocysticercosis. It most often involves the lower spinal cord; one of its earliest signs is low back pain with radiculopathy.

Toxoplasmosis is the most common opportunistic central nervous system infection affecting patients with AIDS. However, spinal cord involvement is not common. Spinal echinococcal disease can be confused with spinal tuberculosis, especially that *Mycobacterium tuberculosis *and *Echinococcus *are present in the same endemic areas. Surgery with laminectomy and decompression is the treatment of choice for spinal echinococcal disease. Needle aspiration carries a significant risk of cystic rupture and dissemination of the parasite and should be avoided [14].

i. Spine Infections in Cerebral Palsy Patients

The infection rate in cerebral palsy patients after scoliosis fusion surgery ranges from 1.1% to 15.2%. Deep infections are caused by gram-negative organisms, whereas superficial infections are caused equally by gram-positive and gram-negative organisms. The most common organisms are *Escherichia coli*, *Pseudomonas aeruginosa*, and gram-positive organisms. Risk factors increasing the rate of infection in cerebral palsy patients include a poor nutritional status, a large curve, the presence of baclofen pump, ventriculoperitoneal shunt, gastrostomy tube, gastroesophageal reflux disease, and increased intraoperative blood loss [36].

Nonoperative management of pediatric spine infection

a. Antibiotic Treatment

Treatment for pediatric spinal infection is usually with empiric intravenous antibiotics. The antibiotics choices in cases presenting clinically with pyogenic spondylodiscitis, without suspicion of an atypical organism, are as follows: (1) for neonates and infants under six months, amoxicillin/clavulanate with gentamicin to cover methicillin-sensitive Staph, *Streptococcus agalactia*, and gram-negative bacteria; (2) for infants between six months and four years, amoxicillin/clavulanate or cefuroxime to cover *Kingella kingae*; and (3) for children over four years, flucloxacillin or amoxicillin/clavulanate to cover MSSA [2].

Methicillin-resistant *S. aureus*, should also be considered, Hawkins et al. found six out of nine of their cases of spinal epidural abscess to grow methicillin-resistant *S. aureus*, possibly indicating the changing prevalence and invasiveness of community-acquired MRSA [31].

Spinal epidural abscesses have been traditionally treated with surgical decompression and drainage in combination with antibiotics [31], although recent literature suggests that surgery can be avoided in favor of antibiotic therapy, in combination with other nonoperative treatment modalities like minimally invasive drainage techniques [31,32].

For patients with spinal tuberculosis, treatment includes isoniazid (5-10 mg/kg), rifampicin (10-20 mg/kg), ethambutol in children older than six years (15 mg/kg), and pyrazinamide (25 mg/kg) for one year [37].

For patients with the rare epidural abscess complicating Lemierre disease, the causative organism *Fusobacterium necrophorum* will need treatment with IV meropenem for six weeks in addition to surgery [12].

Patients with diagnosed HSV sacral myeloradiculitis have been reported to achieve complete resolution of symptoms after three weeks of IV acyclovir [7].

b. Interventional Treatment

CT-guided biopsy is reserved for cases resistant to antimicrobial therapy or when there are concerns regarding other pathology mimicking infection, due to its low rate of positive cultures [4]. In instances that a CT-guided biopsy is indicated, positive cultures have been shown to reliably provide helpful information in guiding antibiotic treatment [23]. Cases of spinal infection with uncommon causes may require lumbar puncture in order to perform cerebrospinal fluid analysis [7].

Operative management of pediatric spine infections

Although rare, some cases of pediatric spinal infections do require surgical intervention, and those cases are crucial to identify. Generally, surgery is reserved for patients displaying progressive neurologic deficit or progressive instability [38]. The indications for decompression and atlantoaxial fusion for cervical spine tuberculosis in children are neurologic deficit, atlantodental interval greater than 5 mm on flexion/extension views, and progressive deformity [37].

In pediatric spinal tuberculosis throughout the spinal column for patients with active tuberculosis, posterior instrumented stabilization combined with anterior radical debridement should be reserved only for advanced tuberculosis with instability, rapid progress of kyphosis, and unacceptable pre-existing kyphosis [27].

A novel surgical treatment of a three-month-old infant with vertebral osteomyelitis displaying neurological decline from progressive instability of the occiput, C1, and C2 levels after failed bracing was described [38]. The patient had reduction and occiput to C2 posterior fusion and segmental instrumentation, with nonsegmental instrumentation extending in the soft tissues from C2 to T4 with the intent of providing stabilization without fusion and permitting further growth. This technique was used in order to overcome the difficulty of providing secure immobilization of the craniocervical junction while not creating an inadvertent fusion of the sub-axial cervical spine.

Complications of pediatric spine infections

Children with spinal infections generally have good functional outcomes after completing treatment, in part due to their remodeling potential [37]. Poor outcomes, however, do unfortunately occur. Many of these complications are seen in patients that undergo operative management, but some are seen in patients managed conservatively as well. Patients with a history of spondylodiscitis can have persistent intermittent back pain usually with little to no impact on daily activities [4]. Some patients with spondylodiscitis of the cervical spine, however, can develop auto fusion or deformity of adjacent vertebrae leading to a significant decrease in neck range of motion and subsequent debility [24]. Patients with spondylodiscitis of the thoracic or lumbar spine are at the risk of developing scoliosis or kyphosis, sometimes requiring surgical correction [7,24]. Glotzbecker et al. noted in their case report of neonatal axial spine osteomyelitis that the patients continued to have mild global developmental delay two years following successful hardware removal [38]. Historical literature has shown that non-surgical management of spinal infections in children frequently leads to paraplegia [27] and was routinely fatal [30], but the adoption of modern practices has led to massive improvements in both morbidity and mortality.

Differential diagnosis of pediatric spine infection

Conditions to be considered in the differential diagnosis in a child presenting with back pain and a lytic lesion of the spine include eosinophilic granulomas, leukemia, lymphoma, fibrous dysplasia, and Ewing's sarcoma.

## Conclusions

Albeit a rare occurrence in clinical practice, having a low threshold of suspicion is needed when faced with possible pediatric spine infection as early recognition and prompt initiation of appropriate treatment are paramount in achieving a satisfactory resolution of the disease. Invasive diagnostic procedures should be considered in atypical and chronic cases as well as in cases not responding to IV antibiotics. Other differential diagnoses that are common which the physicians should be aware and exclude efficiently includes eosinophilic granulomas, leukemia, lymphoma, fibrous dysplasia, or Ewing's sarcoma.
